# Differences in physical activity time-use composition associated with cardiometabolic risks

**DOI:** 10.1016/j.pmedr.2018.11.006

**Published:** 2018-11-13

**Authors:** D.E. McGregor, J. Palarea-Albaladejo, P.M. Dall, E. Stamatakis, S.F.M. Chastin

**Affiliations:** a Institute for Applied Health Research, Glasgow Caledonian University, Glasgow, Scotland, UK; b Biomathematics and Statistics Scotland, JCMB, The King's Buildings, EH9 3FD Edinburgh, UK; c Charles Perkins Centre, Epidemiology Unit, School of Public Health, University of Sydney, Sydney, Australia; d Department Movement and Sports Sciences, Ghent University, Ghent, Belgium

**Keywords:** MVPA, Sedentary behavior, Physical activity, Compositional data analysis, Cardiometabolic health, Adipoisity

## Abstract

This study investigates the association between the overall physical activity composition of the day (sedentary behavior (SB), light intensity physical activity (LIPA) and moderate-to-vigorous physical activity (MVPA)) and cardiometabolic health, and examines whether improved health can be associated with replacing SB with LIPA. A cross-sectional analysis of the Health Survey for England 2008 on N = 1411 adults was undertaken using a compositional analysis approach to examine the relationship between cardiometabolic risk biomarkers and physical activity accounting for co-dependency between relative amounts of time spent in different behavior. Daily time spent in SB, LIPA and MVPA was determined from waist-mounted accelerometry data (Actigraph GT1M) and modelled against BMI, waist circumference, waist-to-hip ratio, blood pressure, total and HDL cholesterol, HbA1c, and VO_2_ maximum. The composition of time spent in SB, LIPA and MVPA was statistically significantly associated with BMI, waist circumference, waist-to-hips ratio, HDL cholesterol and VO_2_ maximum (p < 0.001), but not HbA1c, systolic and diastolic blood pressure, or total cholesterol. Increase of relative time spent in MVPA was beneficially associated with obesity markers, HDL cholesterol, and VO_2_ maximum, and SB with poorer outcomes. The association of changes in LIPA depended on whether it displaced MVPA or SB. Increasing the proportion of MVPA alone may have the strongest potential association with adiposity outcomes and HDL cholesterol but similar outcomes could also be associated with a lower quantity of MVPA provided a greater quantity of SB is replaced overall with LIPA (around 10.5 min of LIPA is equivalent to 1 min of MVPA).

## 1 Introduction

The benefits of physical activity for cardiometabolic health are widely accepted (Andrade and Ignaszewski, 2007). Yet while public engagement in sport and exercise has remained relatively stable over time, levels of domestic and occupational physical activity have declined and time spent sedentary (sitting) has risen significantly (Ng and Popkin, 2012). Greater time spent in sedentary behavior (SB) necessarily displaces physical activity, and more recent studies have reported evidence for deleterious effects of the actual amount of SB time itself (Biswas et al., 2015), beyond the impact of displacing other daily physical activity behaviors.

Several current national guidelines now recommend to decrease time spent in SB in addition to engaging in daily moderate to vigorous activity (MVPA) (Department of Health, 2011; The Australian Government, 2014), while some advocate promotion of leisure time MVPA only (Ekelund et al., 2016a; van der Ploeg and Hillsdon, 2017). It was shown that 60–75 min of daily MVPA is required to negate the effect of too much time spent in SB, but conversely the results also suggest that sitting over 8 h per day can negate the beneficial effect of MVPA for people who just meet the recommended guidelines (equivalent to 150 min of moderate intensity activity per week) (Ekelund et al., 2016a). In addition, 60–75 min of daily MVPA may not be possible for some, or easily achievable for a large portion of the population. In some cases this is due to physical limitations, but lack of time is the most commonly cited barrier to exercise and MVPA in adults (Booth et al., 1997), suggesting greater consideration be given to the role of time spent in light intensity physical activity (LIPA), generally incidental to daily living. This is in line with the recent report by the 2018 Physical Activity Guidelines Advisory Committee in the United States which noted that the benefits of physical activity can be achieved in a variety of ways, and highlighted that the role of time spent in light intensity physical activity (LIPA) has yet to be elucidated (Young et al., 2016; US Department of Health and Human Services, 2018).

As the time available each day is finite, regardless of whether we consider the 24-h cycle or waking day, change in time spent in one behavior will necessarily impact time spent in the other behavior (Buman et al., 2014; Chastin et al., 2015). Therefore, to understand which daily time-uses are associated with better health, it is necessary to determine how health outcomes are associated with the overall daily composition of behaviors, and then explore the implied associations of displacing time from one behavior to the other.

The aims of this paper were to investigate the combined association of time spent in SB, LIPA and MVPA with cardiometabolic health markers using compositional analysis (Chastin et al., 2015), and to estimate time-use behavioral modifications associated with more favorable cardiometabolic health markers.

## 2 Methods

### 2.1 Design

This study used the 2008 Health Survey for England (HSE) data. The Health Survey for England is a series of annual surveys designed to measure health and health-related behaviors in a representative sample of adults and children living in private households in England. The 2008 survey included objective assessment of physical activity for a sub-sample of participants. Ethical approval for the 2008 survey was obtained from the Oxford A Research Ethics Committee (reference number 07/H0604/102) and includes informed consent for all participants. The methods and procedures employed in the 2008 survey are detailed in the HSE Report (Aresu et al., 2008).

### 2.2 Participants

The analysis was restricted to adult participants (21 to 64 years old inclusive) with valid accelerometry data (Aresu et al., 2008). In particular, individuals with less than 4 days valid accelerometry data were excluded from the analysis. Individuals with missing covariates and biomarker data were also excluded.

Assessment of composition of the waking day

Time spent in SB, LIPA and MVPA was assessed objectively in a sub-sample of the survey following the protocol set out in the HSE report (Aresu et al., 2008), using an accelerometer (Actigraph GT1M; Actigraph, LLC, Pensacola, FL). This device was worn on the hip for seven days during waking hours. These data, which consist of acceleration counts integrated over 1-minute epochs, were processed according to standard quality assurance procedures (Esliger and Tremblay, 2007; Esliger et al., 2005). Days when the accelerometer was worn for at least 10 h were considered valid. Each minute epoch was classified using count per minutes thresholds as SB (<200 cpm), LIPA (200 to 2019 cpm) or MVPA (>2020 cpm) (Aresu et al., 2008). A threshold of SB (<100 cpm) is more commonly used, however there is some evidence that the performance of the two thresholds is comparable (Kozey-Keadle et al., 2011) so we have retained the thresholds used by the HSE.

Minutes spent in each of these three behaviors were then totaled over all available valid days (by individual) and converted into an average day for an individual, expressed as proportions of waking day.

### 2.3 Outcomes

The cardiometabolic risk markers considered were body mass index (BMI), waist circumference, waist-to-hip-ratio, maximum oxygen consumption (VO2), high-density lipoprotein (HDL) and total cholesterol levels, systolic and diastolic blood pressure, and glycated hemoglobin (HbA1c). These were measured as described in the supplementary materials. Full details can be found on the NHS Digital Website (Aresu et al., 2008).

### 2.4 Statistical analysis

This is a cross-sectional study, where data modelling was conducted using the compositional statistical methodology (Chastin et al., 2015) applied to time-use during the waking day across three distinct physical activity behaviors: SB, LIPA, and MVPA.

The proportions of the waking day spent in (SB, LIPA, MVPA) must total to 1 by definition, therefore this composition can be reduced to 2 variables with no loss of information. In principle, this could be any two components with no alterations, however co-dependency between the components makes regression carried out using these components potentially misleading. For example, basing the regression analysis on (SB, LIPA) only would still incorporate the information contained in the MVPA component - it is therefore incorrect to attribute differences associated with these two components to SB and LIPA only.

The solution is to construct two orthonormal coordinates which can each be interpreted in isolation. One such construction is the isometric log-ratio (ilr) transformation (Egozcue et al., 2003). A particular system of ilr-coordinates was used in our regression models which highlighted the importance or dominance of one behavior relative to an average of the other two, respectively placed in the numerator and denominator of the log-ratio term of the first ilr-coordinate (Hron et al., 2012). The residual information in the composition is captured in the log-ratio of the two components on the denominator, and forms the second ilr-coordinate. Thus we obtain:

(1) $${\mathrm{ILR}}_{1}^{1}=\sqrt{\frac{2}{3}}\ln\frac{\mathrm{LIPA}}{\sqrt{\mathrm{MVPA}\cdot \mathrm{SB}}}\text{and}$$

(2) $${\mathrm{ILR}}_{2}^{1}=\sqrt{\frac{1}{2}}\ln\frac{\mathrm{SB}}{\sqrt{\mathrm{MVPA}}}$$

where (*SB*, *LIPA*, *MVPA*) are the proportions of the waking day spent performing each behavior type, the first coordinate (*ILR*_1_^1^) is useful for assessing the relationship between the relative importance of LIPA in the time-use composition and cardiometabolic health outcomes.

The expected outcome can then be modelled as Model 1:

(3) $$Y=\;\gamma_{1}^{1}{\mathrm{ILR}}_{1}^{1}+\gamma_{2}^{1}{\mathrm{ILR}}_{2}^{1}+\beta^{T}x+\varepsilon$$

where *Y* is the cardiometabolic risk marker considered, *γ*_1_^1^ and *γ*_2_^1^ are the regression coefficients of the ilr-coordinates, *x* is the vector of confounding variables with associated regression coefficients *β*, and *ε* is the ordinary normally distributed random error term.

It is possible to describe the same time-use composition using alternative ilr-coordinates so that information about the relative importance of a different behavior is isolated instead. For instance, the relative importance of SB was isolated by using Eqs. (4), (5) to define Model 2 below (Eq. (6)):

(4) $${\mathrm{ILR}}_{1}^{2}=\sqrt{\frac{2}{3}}\;\ln\frac{\mathrm{SB}}{\sqrt{\mathrm{MVPA}\;\mathrm{LIPA}}}\text{and}$$

(5) $${\mathrm{ILR}}_{2}^{2}=\sqrt{\frac{1}{2}}\ln\frac{\mathrm{LIPA}}{\mathrm{MVPA}},$$

(6) $$Y=\gamma_{1}^{2}{\mathrm{ILR}}_{1}^{2}+\gamma_{2}^{2}{\mathrm{ILR}}_{2}^{2}+\beta^{T}x+\varepsilon$$

It is important to note that Model 2 has obviously different coefficient estimates *γ*, but it has the same statistical characteristics and produces the same predictions as Model 1 (Eq. (3)) (see supplementary materials for further details). These methods are described in more detail elsewhere (Egozcue et al., 2003; Hron et al., 2012). The use of log-ratios introduces some complexity into the interpretation of the results. It is then useful to seek for the simplest representation of the composition in terms of ilr-coordinates associated with an outcome. We did this by using likelihood ratio tests (LRT) to eliminate from the models those ilr-coordinates not being statistically significant, leading to models that were easier to interpret. These simplified models were then used to forecast each cardiometabolic risk marker given different initial time-use compositions and estimate the changes in physical activity time composition associated with a given change in health outcome.

Confounding variables were assessed for inclusion in the models using stepwise linear regression and backwards elimination based on LRT and the Akaike Information Criteria (AIC) (Akaike, 1998). More specifically, at each step, variables with an associated p-value > 0.2 were considered for elimination following (Budtz-Jørgensen et al., 2007), and the one which minimized the AIC value on elimination was selected. The following variables were considered for inclusion: age, sex, marital status, ethnic origin, highest educational attainment, low household income (<£2600 pa), greatest daily alcohol consumption in last week, number of days consumed alcohol in last week, currently taking cardiovascular medicine, smoker status, previous diagnosis of cancer, previous diagnosis of stroke or cardiovascular problems, previous diagnosis of diabetes. Details of the confounding variables included for each outcome are provided in the Supplementary Materials. (Inclusion of the confounding variables was confirmed independently with reference to whether a plausible causal path existed, and additional sensitivities such as re-ordering of the variables were carried out manually. In each case, the results of variable selection were found to be broadly reasonable and retained. In addition, interactions between age and the confounding variables were tested for, and no strong support was found for interactions of this kind (p-value > 0.05 for all cases).)

Ordinary regression model assumptions were examined, alongside the coefficient of determination (R^2^). Statistical significance of the model coefficients was concluded for p-values below the usual 0.05 significance level. In accordance with STROBE guidelines, a sensitivity analysis was conducted for each model by removing 10% of cases at random and checking for a statistically significant change in the results (von Elm et al., 2007). No statistically significant changes were found. All data analyses and graphical representations were conducted using the R statistical system version 3.4.1.

Six individuals had no MVPA time, and these zeroes were imputed by using the log-ratio EM algorithm (Palarea-Albaladejo and Martín-Fernández, 2008; Palarea-Albaladejo and Martín-Fernández, 2015). This allowed the calculation of log-ratios involving MVPA for those individuals while, importantly, the log-ratios between the other behaviors were preserved.

The full methodological procedure is described in detail in the supplementary materials.

## 3 Results

**Fig. 1:**
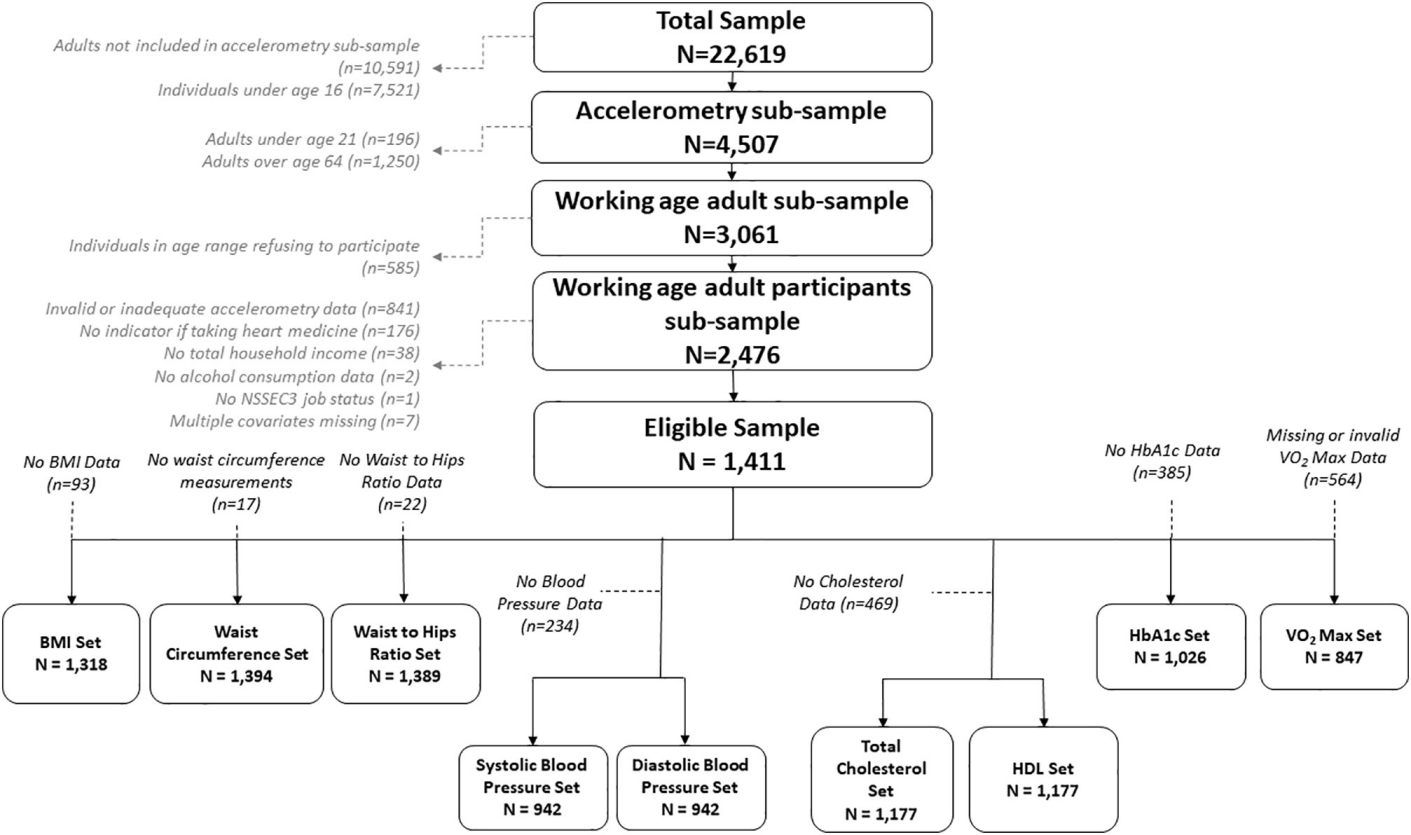
Consort diagram illustrating flow of data in this study using Health Survey for England 2008.

The complete HSE 2008 survey included 22,619 individual records. A subsample of N = 4507 adults (over 16 years) were asked to wear an accelerometer, and of these, 3061 were within the age range of this study. This study's analysis dataset comprises of 1411 of these individuals who had valid accelerometry data and the full set of covariates. The data set analyzed has similar demographic characteristics to the survey population (as summarized and compared in the supplementary materials). The data flow is illustrated in Fig. 1.

Results of linear regression models for cardiometabolic risk markers are presented in Table 1. BMI, waist circumference, waist-to-hips ratio, and HDL cholesterol were all best described by the ilr-coordinate in Eq. (2) whereas VO_2_ maximum was best described by the ilr-coordinate in Eq. (4) (p-value > 0.05 for all outcomes in LRT compared to the full model including the two ilr-coordinates).

**Table 1:** Retained ilr-coordinate, regression coefficients, coefficients of determination and p-values from likelihood ratio test against competing model with no effect of the waking day composition for cardiometabolic risk markers investigated.

Cardio-metabolic risk marker	Retained ilr-coordinate^a^	Regression coefficient^b^	Coefficient of determination (R^2^)^c^	LRT p-value for overall significance of waking day composition^d^
BMI^v^	$\;\;\frac{1}{\sqrt{2}}\ln\frac{\mathrm{SB}}{\mathrm{MVPA}}$	1.374	0.1817	<0.001
Waist circumference^e^	$\;\;\frac{1}{\sqrt{2}}\ln\frac{\mathrm{SB}}{\mathrm{MVPA}}$	3.601	0.3152	<0.001
Waist-to-hips ratio^e^	$\;\;\frac{1}{\sqrt{2}}\ln\frac{\mathrm{SB}}{\mathrm{MVPA}}$	0.011	0.5034	<0.001
HDL^e^	$\;\;\frac{1}{\sqrt{2}}\ln\frac{\mathrm{SB}}{\mathrm{MVPA}}$	−0.050	0.2492	<0.001
Total cholesterol^f^	–	–	–	0.090
Systolic blood pressure^f^	–	–	–	0.467
Diastolic blood pressure^f^	–	–	–	0.199
VO_2_ maximum	$\sqrt{\frac{2}{3}}\ln\frac{\mathrm{SB}}{\sqrt{\mathrm{MVPA LIPA}}}$	−0.075	0.3708	<0.001

a The physical activity ilr-coordinate retained in the simplest model.

b The regression coefficient determines the strength and direction of the association between the outcome and the retained regression coordinate, after allowing for possible confounders. Where the term is positive it indicates the risk marker increases with increases in the ilr-coordinate.

c The coefficient of determination is the proportion of observed variance in the outcome explained by the model.

d The p-value shows the significance of including both ilr-coordinates, and is based on a likelihood ratio test (LRT) between the complete model and a model excluding physical activity entirely.

e Although time spent in LIPA does not appear explicitly in the retained ilr-coordinate for these cardiometabolic risk markers, it is implicitly included in this model as it influences the balance of time between SB and MVPA.

f No statistically significant association was found between these cardiometabolic risk markers and the composition of the waking day so no retained ilr coordinate is specified.

The assumptions underlying the linear regression model were not satisfied for glycated hemoglobin. No statistically significant association between any of the other cardiometabolic risk markers and the composition of the waking day was found (for systolic blood pressure, p-value = 0.467; diastolic blood pressure, p-value = 0.199; and total cholesterol, p-value = 0.090).

**Fig. 2:**
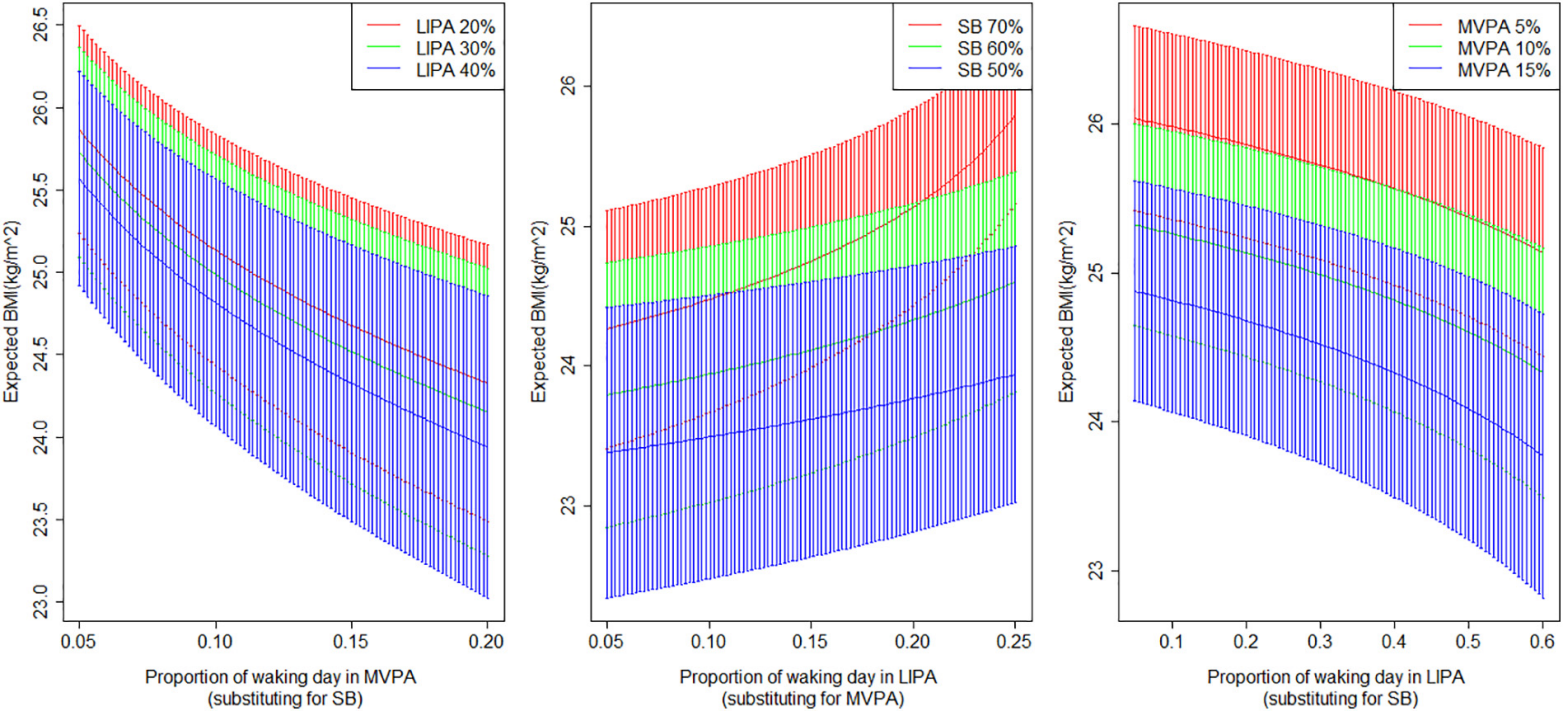
Expected BMI (and 95% confidence intervals) predicted based on the balance of SB and MVPA against different compositions of physical activity in the waking day shown as functions of MVPA substituted for SB depending on different time spent in LIPA, LIPA substituted for MVPA, and LIPA substituted for SB. Based on Health Survey for England 2008.

The estimated coefficients for Eq. (2) show that the increasing proportion of time spent in SB relative to MVPA is associated with higher BMI, waist circumference, and waist-to-hip ratio, and lower HDL cholesterol; whereas increasing the proportion of time spent in MVPA with respect to SB will have the opposite association. Note that time spent in LIPA is implicitly included in this model as it influences the balance of time between SB and MVPA. The association of these cardiovascular markers with greater time spent in LIPA depends on whether the time is reallocated from SB or MVPA. Fig. 2 shows how LIPA is associated with the outcomes through the balance between SB and MVPA for BMI. Similar output is observed for the other cardiometabolic markers modelled by the ilr-coordinate in Eq. (2).

The estimated coefficients for Eq. (4) suggest that a lower proportion of time spent in SB with respect to LIPA or MVPA will tend to be associated with a higher estimated VO_2_ maximum. Note that the impact of a given change in one of the original components on the ilr-coordinate will reduce as the value of the component increases. In this dataset MVPA is significantly lower than LIPA for all individuals so the model does not imply that LIPA and MVPA are interchangeable. However, it still suggests LIPA has a stronger positive association with this outcome than the others.

**Fig. 3:**
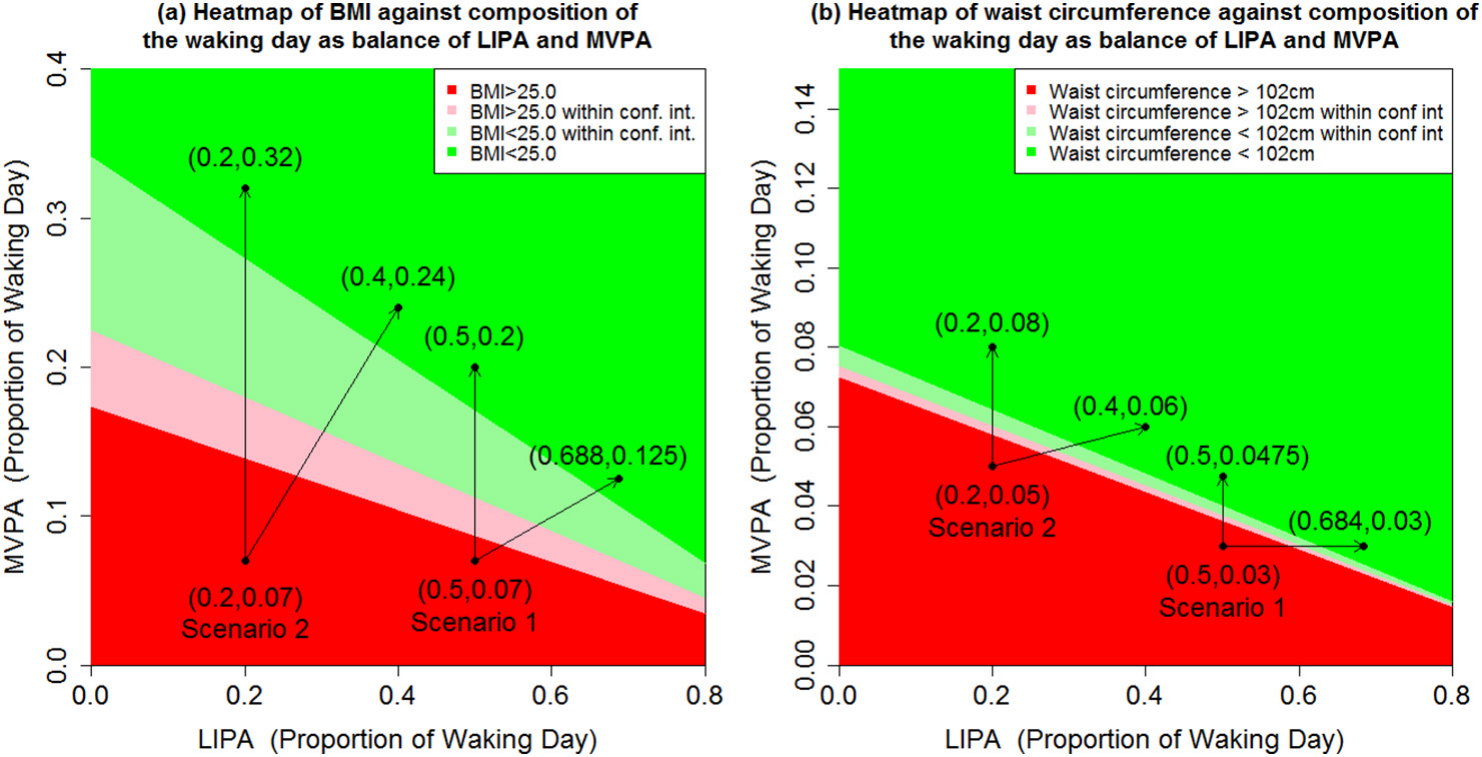
Expected (a) BMI and (b) waist circumference against composition of the waking day according to balance of MVPA and LIPA, with the remaining proportion of the waking day made up of SB, for a white British male with degree level education, not taking heart medicine and (a) aged 40 with no history of diabetes, and living in a household where total income is over £2600pa; (b) aged 40 with no history of diabetes, and living in a household where total income is under £2600pa. The pale regions referred to as within confidence interval are combinations of LIPA and MVPA where the 95% confidence interval for the expected outcome includes the threshold, either BMI = 25.0 kg m^−2^ or waist circumference = 102 cm. Based on Health Survey for England 2008.

**Fig. 4:**
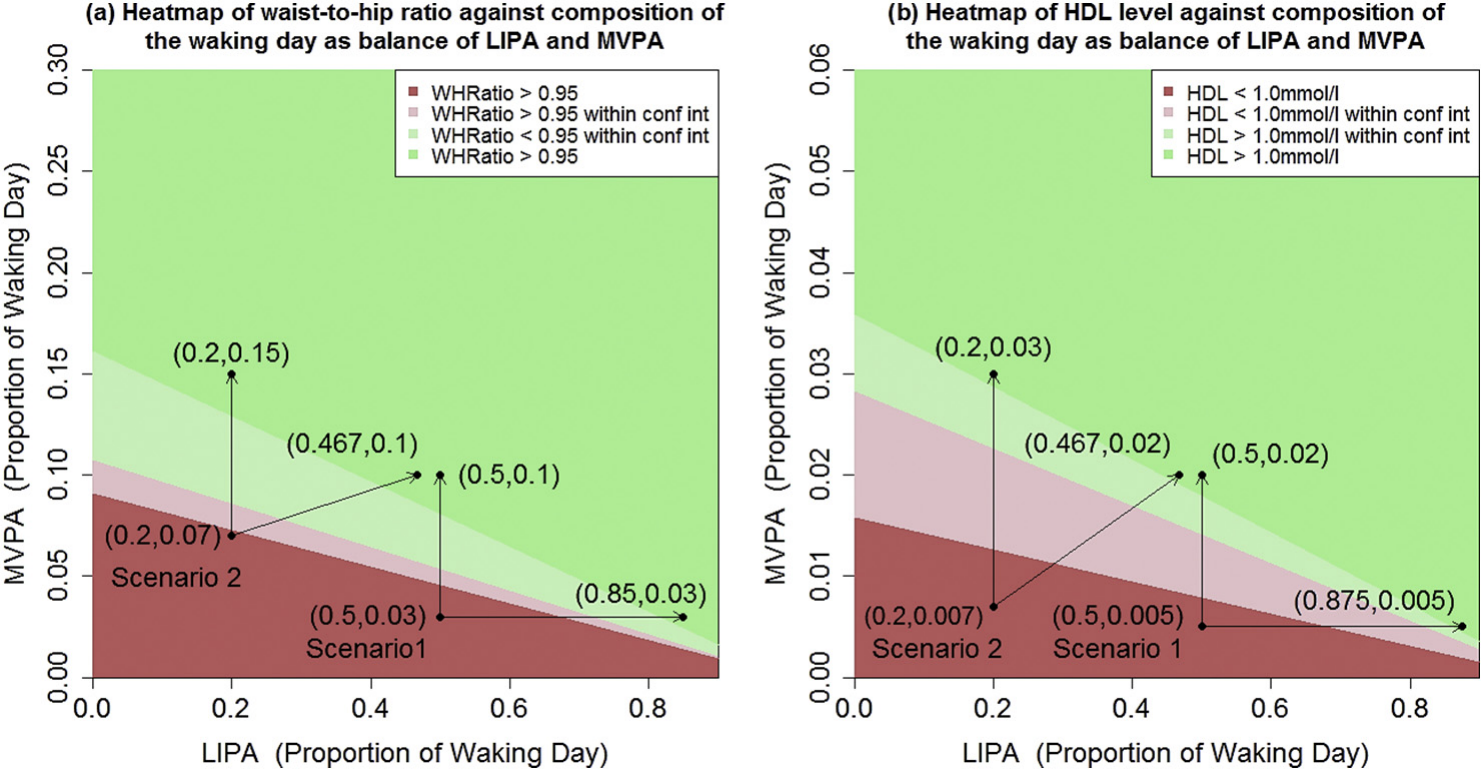
Expected (a) waist-to-hips ratio and (b) HDL cholesterol level against composition of the waking day according to balance of MVPA and LIPA, with the remaining proportion of the waking day made up of SB, for a male with degree level education, maximum daily alcohol consumption ≤ 4 units, no history of cardiovascular problems or stroke, not taking heart medicine and (a) aged 50 with no history of cancer, a previous diagnosis of diabetes, and living in a household where total income is under £2600pa; (b) aged 40 with no history of diabetes, or cancer, and living in a household where total income is under £2600pa. The pale regions referred to as within confidence interval are combinations of LIPA and MVPA where the 95% confidence interval for the expected outcome includes the threshold, either Waist-to-hip ratio = 0.95 or HDL = 1.0 mmol/L. Based on Health Survey for England 2008.

**Fig. 5:**
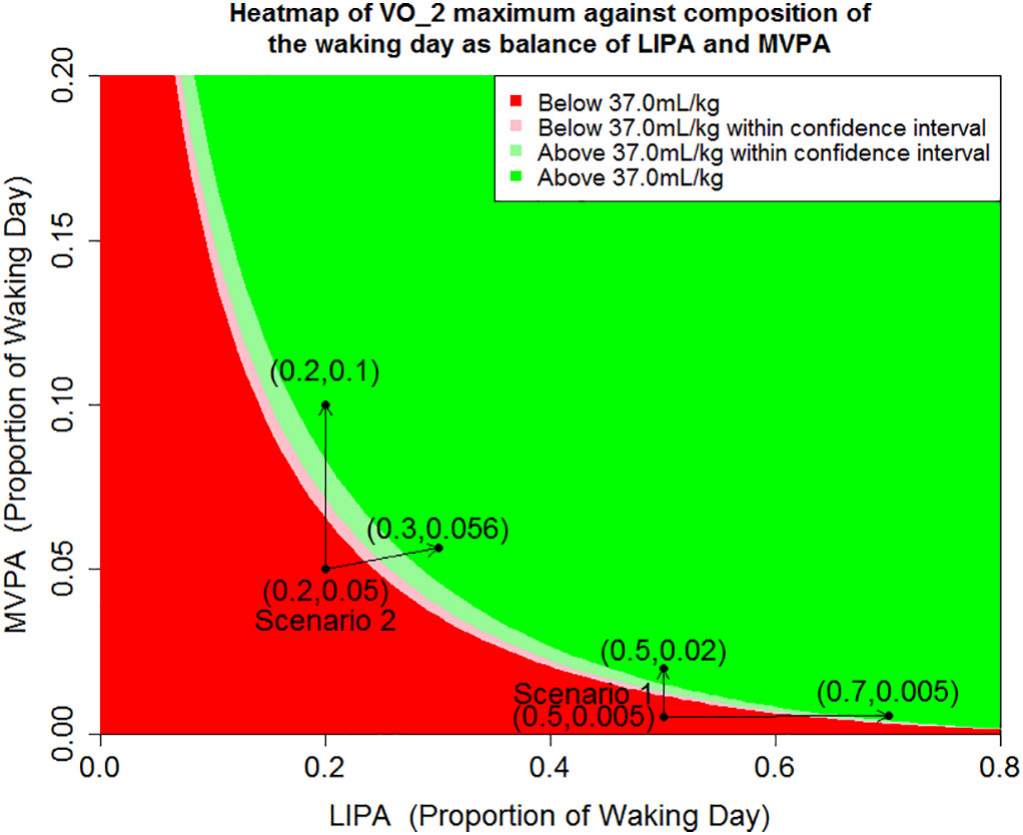
Expected VO_2_ maximum (and 95% confidence intervals) against combinations of MVPA and LIPA (with the remaining proportion of the waking day made up of SB) for a white British male with degree level education not taking heart medicine and aged 40 with no history of diabetes. The pale regions referred to as within confidence interval are combinations of LIPA and MVPA where the 95% confidence interval for the expected outcome includes the threshold VO_2_ maximum = 37.0 mL/kg. Based on Health Survey for England 2008.

The fitted models were then used to predict the expected cardiometabolic risk markers in a set of scenarios representative of different individuals with poor metabolic profiles, and changes in physical activity time composition associated with improved outcomes were identified. These are shown in Fig. 3, Fig. 4, Fig. 5 for BMI, waist circumference, waist-to-hip ratio, HDL cholesterol, and VO_2_ maximum.

Fig. 3(a) provides a heat map of BMI against composition of the waking day according to balance of MVPA and LIPA, with the remaining proportion of the waking day made up of SB. Scenarios 1 and 2 are both associated with being overweight (BMI > 25.0) and having similar levels of MVPA. In scenario 1: MVPA = 7%, LIPA = 50%, SB = 43% (~67 min MVPA, 480 min LIPA and 413 min SB assuming a 16 h waking day). Raising the level of MVPA to 20% (replacing SB exclusively) gives a scenario associated with normalized BMI. In contrast, for scenario 2: MVPA = 7%, LIPA = 20%, SB = 73% (~67 min MVPA, 192 min LIPA and 701 min SB assuming a 16 h waking day) MVPA would need to be raised to around 32% (replacing SB exclusively) to achieve a similar outcome. In both scenarios, similarly beneficial associations were observed by replacing SB with a mixture of LIPA and MVPA. For example, in Scenario 1, increasing MVPA to 13% and LIPA to 69%, is associated with a similar outcome as increasing MVPA to 20% in isolation.

Fig. 5 shows two similar scenarios for VO_2_ maximum, however the differences are starker than in Fig. 3, Fig. 4. We remark that the threshold of 37.0 mL/kg does not have specific clinical significance, but is approximately equal to the upper bound of the third quartile of the VO_2_ maximum data.

## 4 Discussion

The results of this study add to the growing number of recent studies which report that some cardiometabolic outcomes are jointly associated not just with time spent in MVPA or in SB but also with the composition of daily time-use in all movement behaviors (Chastin et al., 2015; Carson et al., 2016; Carson et al., 2017; Dumuid et al., 2016; Ekelund et al., 2016b). The results provide new insights about the synergistic associations of the different behaviors, although MVPA remains the most potent part of this mixture. For example, Fig. 3(b) suggests that each minute of MVPA is associated with a change in outcome around 10.5 times the size of the change associated with each minute of LIPA.

Two recent investigations into the associations of LIPA with health using the isotemporal substitution paradigm (Buman et al., 2014; Hamer et al., 2014) reported larger associations with substitution of MVPA for SB than our analysis, and no significant association with substitution of LIPA for SB.

For our model the associations of MVPA are also dependent on the balance of time spent between LIPA and SB. Similarly, we find that time spent in SB has a detrimental association with cardiometabolic risk markers, but the magnitude of the association depends on the balance of time between MVPA and LIPA. The results imply that the role of LIPA is ambivalent and can be positive for health when it replaces SB but negative if it displaces time spent in MVPA, as shown in Fig. 2.

The heat maps (Fig. 3, Fig. 4, Fig. 5) show that the same improved cardiometabolic outcomes could be attained in multiple ways, supporting recently published research supporting national health guidelines (US Department of Health and Human Services, 2018). They support the widely accepted benefits of MVPA, but also illustrate that simply eliminating SB from the daily routine (replacing it with LIPA) has beneficial associations. For example, in Fig. 3(b), an expected waist circumference (101.5 cm) is associated with a time-use composition of 8% MVPA, 10% LIPA and 82%SB (~77 min MVPA, 96 min LIPA and 787 min SB assuming a 16 h waking day) which is typical of an office worker who spends time in the gym each day. The same expected waist circumference was also associated with a composition of 2.5% MVPA, 72.5% LIPA, 25% SB (~24 min MVPA, 696 min LIPA and 240 min SB), which is more typical of an individual who does not quite meet the recommended MVPA guidelines but spends less time sedentary.

Lastly, we note that the current study also found no statistically significant association between the composition of daily time-use and blood pressure (systolic and diastolic), or total cholesterol. One previous study showed an association between blood pressure and composition of 24 h daily time-use, but the association was driven significantly by the proportion of time spent asleep relative to other behaviors, which likely accounts for the apparent discrepancy (Chastin et al., 2015). Current guidelines (Brook et al., 2013; Pescatello et al., 2004; Whelton et al., 2002) recommend physical exercise as a means to reduce the risk of hypertension, and there is a body of non-compositional analyses supporting this recommendation, although the evidence for a dose-response relation is more mixed (Diaz and Shimbo, 2013). A small number of non-compositional studies, in particular one based on the same dataset (HSE 2008), are in line with the negative result on blood pressure (O'Donovan et al., 2013; Healy et al., 2011; Pereira et al., 1999).

### 4.1 Strengths and limitations

The strength of this study resides in the compositional approach applied to model a well-known data set, in which physical behaviors are measured objectively. The compositional approach produces estimates that are fully adjusted for all time use and provides information about the combined and synergistic associations of the different behaviors.

A limitation of the study is that it is cross-sectional and therefore the estimates and forecasts computed should be interpreted with care. As with all cross-sectional analysis, causal inference is limited and the estimated associations may reflect a population shift in distribution of time rather than actual effects for individuals. This is particularly important for outcomes where reverse causality can play a role, as is known to be the case for adiposity outcomes, and so associations might be inflated. However this does not detract from the fact that different compositions of time use can be associated with the same outcome. This type of compositional analysis should be repeated on longitudinal data to provide evidence of individual change more accurate estimates, and potential causality.

Another limitation is the fact that the Actigraph monitor used in this study cannot discriminate between postural sitting and standing and as such may provide less accurate estimates of LIPA and SB (Kozey-Keadle et al., 2011; Sellers et al., 2016). Therefore, it is possible that the proportion of time allocated to SB and LIPA are not accurate and that SB time has been over estimated. In addition, the threshold between SB and LIPA used is at the high end of the range of thresholds used in studies of this kind, so it is further likely that SB time has been over estimated. Data for sleep time were not available, however this does highlight that composition of the waking day on its own can be used effectively in this type of modelling. The accelerometer derived data also does not provide information on the context under which movement takes place. There may be important differences in the relationship between MVPA and cardiovascular health, for example, if most of the MVPA is done under a strenuous work setting, as opposed to leisure activities. Lastly, we note that the survey data is now 10 years old, however it is the only survey in the Health Survey for England series with objective physical activity data.

The principal finding of our analysis is that an improved cardiometabolic risk profile is associated with a variety of behavioral modifications. Our results add to the current cross-sectional evidence that decreasing inactivity, in particular sedentary time, and maintaining time spent in, or reallocating additional time to, MVPA, are associated with a more favorable cardiometabolic risk profile for adiposity-related outcomes and HDL cholesterol. However, it also demonstrates reallocating time from sedentary behavior to light intensity physical activity (without an increase in MVPA) is also associated with more modestly beneficial outcomes. This suggests that, if our results reflect causal relationships, some of the benefits of moderate to vigorous physical activity might be attainable with larger amounts of light intensity physical activity, offering multiple potential paths to better cardiometabolic health.

## Appendix A Supplementary data

Supplementary material. Image 1
