# Supplementary material for: Differences in physical activity time-use composition associated with cardiometabolic risks
Source: Prev Med Rep. 2018 Nov 13;13:23–9. doi: 10.1016/j.pmedr.2018.11.006 (PMC6240623; doi:10.1016/j.pmedr.2018.11.006)
Supplement: Supplementary file 1 — Supplementary material. [file mmc1.docx]

**Additional details on the analysis underlying “What change in time use composition is associated with lower cardiometabolic risks?”**

**Contents**

1. Data Summary
2. Details of cardiometabolic markers
3. Details of modelling process

- ILR Model
- Model fitting process
- Model simplification process
- Covariates included

1. Sensitivity analysis
2. Codes

**1. Data Summary**

| Characteristic | Valid Accelerometry Data | All Data (Aged 21-65) |
| --- | --- | --- |
| N | 1635 | 10676 |
| Age (median , [IRQ] years) | 46 [35-56] | 43 [34-54] |
| Age Distribution (%) |  |  |
| 21-39 | 34.6 | 38.9 |
| 40-59 | 50.4 | 49.0 |
| 60-64 | 15.0 | 12.1 |
| Sex Distribution (%) |  |  |
| Male | 45.0 | 44.9 |
| Female | 55.0 | 55.1 |
| Marital Status Distribution (%) |  |  |
| Married | 57.6 | 56.3 |
| Widowed | 2.1 | 1.7 |
| Divorced | 7.9 | 7.4 |
| Separated | 3.1 | 2.7 |
| Never married | 13.5 | 17.0 |
| Cohabiting | 15.7 | 14.7 |
| Civil partnership | 0.0 | 0.1 |
| Ethnic Origin Distribution (%) |  |  |
| White - British | 87.7 | 82.7 |
| White - Irish | 0.8 | 1.2 |
| Any other white background | 3.8 | 4.7 |
| Mixed - White and Black Caribbean | 0.4 | 0.4 |
| Mixed - White and Black African | 0.1 | 0.2 |
| Mixed - White and Asian | 0.1 | 0.1 |
| Any other mixed background | 0.4 | 0.4 |
| Asian or Asian British - Indian | 1.8 | 2.9 |
| Asian or Asian British - Pakistani | 0.8 | 1.6 |
| Asian or Asian British - Bangladeshi | 0.4 | 0.6 |
| Any other Asian/Asian British background | 0.9 | 1.0 |
| Black or Black British - Caribbean | 1.1 | 1.0 |
| Black or Black British - African | 1.2 | 1.6 |
| Any other Black/Black British background | 0.1 | 0.1 |
| Chinese | 0.2 | 0.5 |
| Any other | 0.3 | 0.6 |

| Educational Attainment Distribution (%) | | Valid Accelerometry Data | All Data (Aged 21-65) |
| --- | --- | --- | --- |
| NVQ4/NVQ5/Degree or equiv | 27.1 | | 25.0 |
| Higher ed below degree | 12.8 | | 12.3 |
| NVQ3/GCE A Level equiv | 14.9 | | 15.8 |
| NVQ2/GCE O Level equiv | 23.1 | | 23.3 |
| NVQ1/CSE other grade equiv | 3.6 | | 4.6 |
| Foreign/other | 0.6 | | 0.9 |
| No qualification | 17.9 | | 17.7 |
| NVQ4/NVQ5/Degree or equiv | 27.1 | | 25.0 |
| Household Income Distribution (%) | **Valid Accelerometry Data** | | **All Data (Aged 21-65)** |
| Less than £520 | 0.2 | | 0.1 |
| £520 - £1,600 | 0.2 | | 0.2 |
| £1,600 - £2,600 | 0.4 | | 0.3 |
| £2,600 - £3,600 | 0.9 | | 0.7 |
| £3,600 - £5,200 | 1.2 | | 1.3 |
| £5,200 - £7,800 | 2.8 | | 2.8 |
| £7,800 - £10,400 | 3.2 | | 3.3 |
| £10,400 - £13,000 | 3.7 | | 3.6 |
| £13,000 - £15,600 | 3.1 | | 3.5 |
| £15,600 - £18,200 | 5.8 | | 4.3 |
| £18,200 - £20,800 | 3.6 | | 3.8 |
| £20,800 - £23,400 | 5.1 | | 4.3 |
| £23,400 - £26,000 | 3.5 | | 3.5 |
| £26,000 - £28,600 | 3.2 | | 3.0 |
| £28,600 - £31,200 | 3.9 | | 3.5 |
| £31,200 - £33,800 | 3.4 | | 3.4 |
| £33,800 - £36,400 | 4.1 | | 2.9 |
| £36,400 - £41,600 | 5.7 | | 5.3 |
| £41,600 - £46,800 | 5.0 | | 4.5 |
| £46,800 - £52,000 | 5.5 | | 5.6 |
| £52,000 - £60,000 | 5.9 | | 5.1 |
| £60,000 - £70,000 | 4.4 | | 3.6 |
| £70,000 - £80,000 | 3.2 | | 3.2 |
| £80,000 - £90,000 | 1.5 | | 1.6 |
| £90,000 - £100,000 | 0.9 | | 1.5 |
| £100,000 - £110,000 | 1.2 | | 1.1 |
| £110,000 - £120,000 | 0.9 | | 0.6 |
| £120,000 - £130,000 | 0.3 | | 0.5 |
| £130,000 - £140,000 | 0.2 | | 0.3 |
| £140,000 - £150,000 | 0.1 | | 0.3 |
| Greater than £150,000 | 1.0 | | 1.1 |
| Do not know | 5.4 | | 7.7 |
| Refused to answer | 7.6 | | 10.8 |

| Cardiometabolic risk markers |  |
| --- | --- |
| BMI (median [IQR]) kg/m^2^ | 26.71 [24.00-30.31] |
| Waist circumference (median [IQR]) cm | 92.25 [82.21-101.99] |
| Waist to hip ratio (median [IQR]) | 0.8711 [0.8023-0.9371] |
| Systolic Blood Pressure (median [IQR]) mmHg | 125.84 [117.38-136.18] |
| Diastolic Blood Pressure (median [IQR]) mmHg | 73.65 [67.51-80.74] |
| HDL (median [IQR]) mmol/L | 1.5 [1.3-1.7] |
| Total Cholesterol (median [IQR]) mmol/L | 5.6 [4.8-6.3] |
| Glycated Hemoglobin (median [IQR]) % | 5.5 [5.3-5.7] |
| VO_2_ Maximum (median [IQR]) ml/kg | 32.36 [28.42-37.10] |

**2. Details of Cardiometabolic Markers**

The cardiometabolic risk markers considered were BMI, waist circumference, waist-to-hip-ratio, VO2 maximum, HDL levels, total cholesterol levels, systolic and diastolic blood pressure, and glycated hemoglobin.

The measurements of height and weight underlying BMI were taken by interviewers using a portable stadiometer with a sliding head plate, a base plate and three connecting rods marked with a metric measuring scale, and (Soehnle, Seca or Tanita) electronic scales respectively. The measurements of waist and hip circumference were taken by survey nurses using a measuring tape – two measurements were taken and if they were found to differ by more than 3cm a third measurement was taken. We have used the mean of all available measurements in our analysis.

Blood pressures were measured with the Omron HEM907, an oscillometric automated device. Three blood pressure readings were taken, at one-minute intervals, using an appropriately sized cuff on the right arm, with the participant in a seated position after five minutes’ rest. Participants were excluded if they were pregnant. The blood pressure variables used are the means of the second and third measurements obtained from the participants in whom three readings were successfully obtained, excluding those who had eaten, drunk alcohol, exercised, or smoked in the 30 minutes before the measurement was taken.

Two non-fasting blood samples were collected from adults aged 16 and over by survey nurses, and dispatched to the Royal Victoria Infirmary (RVI) Newcastle for analysis. Measurement of total cholesterol was carried out in the Biochemistry Department at the RVI using a Cholesterol Oxidase assay method on an Olympus 640 analyzer calibrated to the Centre for Disease Control (CDC) guidelines. HDL-cholesterol analysis was carried out in the Biochemistry Department at the RVI using a direct method (no precipitation) on an Olympus 640 analyzer. Total glycated hemoglobin (HbA1c) was carried out in the Biochemistry Department at the RVI using the Tosoh G7 analyzer, which was calibrated using Diabetes Control and Complications Trial (DCCT) standards.

Physical fitness among adults aged 16-74 was assessed based on an indirect measure of VO_2_ maximum using a step test to provide an objective measure of physical fitness. The test involved the subject stepping up and down a single step. The pace of stepping increased through the duration of the test. The participant’s heart rate was the primary outcome measure of the step test. The heart rate was recorded at 30 second intervals during the test and at 15 second intervals for two minutes after the step test ended. The participant wore a Polar heart rate monitor round the chest which transmitted the heart rate to a receiver worn on the participant’s wrist. Using a stop watch to mark the time intervals, the nurse recorded the heart rate detected by the monitor. These heart rate measurements were then combined with the resting heart rate obtained earlier during blood pressure measurement to determine the submaximal relationship between heart rate and oxygen uptake. This relationship was then extrapolated up to age-predicted maximal heart rate to provide an estimate of the individual’s maximal oxygen uptake, the overall level of fitness. The method was developed from that devised by researchers in the MRC Epidemiology Unit at Cambridge (1).

More details on all of these measurements are available in the HSE 2008 reports (2).

1. Brage S, Brage N, Franks PW, Ekelund U, Wareham NJ. Reliability and validity of the combined heart rate and movement sensor Actiheart. Eur J Clin Nutr [Internet]. 2005 Apr 16 [cited 2017 Dec 19];59(4):561–70. Available from: http://www.ncbi.nlm.nih.gov/pubmed/15714212

2. Aresu M, Bécares L, Brage S, Chaudhury M. Health Survey for England 2008: Physical Activity and Fitness [Internet]. Vols. 1–2. 2008 [cited 2017 Nov 22]. Available from: https://digital.nhs.uk/catalogue/PUB00430

**3. Details of Modelling Process**

(a) ILR Regression Model

We have used isometric log-ratio (ilr) transformations of the compositional variables in our linear regression models. They allow for an isometric mapping between the simplex of D-part compositions and the (D-1) -dimensional real space (which means that the relative positions of the data points are preserved from the simplex to the real space). That is, if the original composition consists of 3 parts, then we obtain 2 ilr-transformed variables (so-called ilr-coordinates). These new variables are real-valued and as such we can apply standard statistical tools on them. There are infinitely many ilr transformations, which are technically orthogonal rotations of each other, however one particularly convenient for our analysis computes the ilr-variables $z_{1}$ and $z_{2}$ from a 3-part composition $(SB, LIPA, MPVA)$ as:

$$z_{1}= \sqrt{\frac{2}{3}} \ln\frac{LIPA}{\sqrt{MVPA \cdot SB}}, z_{2}= \frac{1}{\sqrt{2}}\ln\frac{SB}{MVPA}$$

This ilr representation isolates in the first ilr-coordinate $z_{1}$ the relative importance or dominance of one component (LIPA above) with respect to the (geometric) average of the other components (MVPA and SB above) and facilitates interpretability. By orthogonal rotation we can alternatively isolate any of the other components in an analogous way using

$$z_{1}= \sqrt{\frac{2}{3}} \ln\frac{SB}{\sqrt{MVPA \cdot LIPA}}, z_{2}= \frac{1}{\sqrt{2}}\ln\frac{LIPA}{MVPA}$$

or

$$z_{1}= \sqrt{\frac{2}{3}} \ln\frac{MVPA}{\sqrt{LIPA \cdot SB}}, z_{2}= \frac{1}{\sqrt{2}}\ln\frac{SB}{LIPA}$$

We can then model an outcome *Y* in terms of the composition simply as

$Y= \gamma_{1}z_{1} + \gamma_{2}z_{2}+ \beta^{T}x+\varepsilon$,

where $\gamma$ are the regression coefficients of the ilr-coordinates representing the original composition, $x$ is a vector of confounding variables, $\beta$ is the vector of regression coefficients associated to them, and $\varepsilon$ is the ordinary normally distributed random error term. This model accounts for all portions of time spent in each (measurable) behavior that add up to a finite time. It therefore accounts for the combined effect of all parts of the composition. It does not matter in what order the parts are transformed (which rotation is selected) as the model gives the same fit with identical coefficient of determination R^2^, p-value for the model and coefficient for the intercept. The interpretation of the common measures associated to the model fitting is the same as for the standard model. The R^2^ coefficient tells us how much of the variance in the outcome is explained by the composition, the p-value for the model tells us if it is a statistically significant model.

Further details about the isometric log-ratio regression model can be found in:

Chastin SFM, Palarea-Albaladejo J. Concise Guide to Compositional Data Analysis for Physical Activity, Sedentary Behavior and Sleep Research: Supplementary Material S2, in Chastin SFM, Palarea-Albaladejo J, Dontje ML, Skelton DA. Combined Effects of Time Spent in Physical Activity, Sedentary Behaviors and Sleep on Obesity and Cardio-Metabolic Health Markers: A Novel Compositional Data Analysis Approach. PLoS One. 2015;10(10):e0139984.

**3. Details of Modelling Process**

(b) Model Fitting Process

An initial model of the form:

$Y= \beta^{T}x+ \varepsilon$ [1]

where $x$ is a vector of the non-compositional confounding covariates, and $\varepsilon$ is a normally distributed error term, was fitted to the data using the usual least squares methodology as a starting point. The objective at this stage was to account for as much of the observed variation as possible without allowing for composition of physical activity in the waking day.

The model was then simplified by a process of backwards elimination. Covariates were considered candidates for elimination if the p-value of the likelihood ratio test (LRT) between the current model and the model omitting the covariate was p > 0.2. Within that constraint we proceeded by optimizing the model with respect to the Akaike Information Criterion (AIC), and stopped when further elimination either failed to improve the AIC or the LRT indicated the variable was significant at the 20% level.

Having eliminated covariates we deemed irrelevant we then proceeded to fit a model of the form:

$Y= \gamma_{1}z_{1} + \gamma_{2}z_{2}+ \beta^{T}x+\varepsilon$ [2]

where $z_{1}$ and $z_{2}$ are the ilr-coordinates obtained from the composition of the waking day, and assessed the significance of the model including the composition of physical activity in the waking day (Eq. 2) based on a LRT with respect to the model excluding physical activity entirely (Eq. 1).

The goal of such a process is to determine the association of the composition of the waking day with the outcome whilst limiting the risk of attributing to physical activity associations that are actually the result of confounding covariates.

(c) Model Simplification Process

The three rotations set out in Section 2 (a) specify equivalent models, however they provide a different breakdown of the association between the cardiovascular risk marker and the composition of the waking day.

Following the principle of parsimony, preference is given to a model with fewer parameters. Thus, for each rotation, we experimented with eliminating each of the regression coefficients ($\gamma_{1}$ and $\gamma_{2}$) in isolation, and compared the full model and the model with one coefficient eliminated by LRT to assess whether the difference between the models was statistically significant. Where the LRT did not show a statistically significant difference we concluded that it was possible to simplify the model by eliminating the corresponding ilr-coordinate.

Our preferred models were based both on the number of parameters in the simplified model and on the reasonability of the physical meaning of the model.

**3. Details of Modelling Process**

(d) Confounding Covariates Included

| **Covariate** | **BMI** | **Waist Circumference** | **Waist--to-hips Ratio** | **Total Cholesterol** | **HDL** | **Systolic blood pressure** | **Diastolic blood pressure** | **VO2 Maximum** | **Glycated Haemoglobin** |
| --- | --- | --- | --- | --- | --- | --- | --- | --- | --- |
| Age | x | x | x | x | x | x | x | x | x |
| Sex | x | x | x |  | x | x | x | x | x |
| Marital Status |  |  |  | x |  |  |  |  |  |
| Ethnic Origin | x | x |  |  |  |  |  |  | x |
| Highest Educational Attainment | x | x | x |  |  | x | x | x | x |
| Low Household Income (<£2,600 pa) | x | x | x |  | x |  | x |  |  |
| Greatest Daily Alcohol Consumption in last week |  |  |  |  | x | x | x |  |  |
| Number of Days consumed alcohol in last week |  |  |  |  |  |  |  |  | x |
| Currently taking cardiovascular medicine | x | x | x | x | x | x | x | x | x |
| Smoker | x | x | x | x | x |  |  |  | x |
| Previous diagnosis of cancer |  |  | x |  | x | x |  |  |  |
| Previous diagnosis of stroke or cardiovascular problems |  |  | x |  |  | x | x |  |  |
| Previous diagnosis of diabetes | x | x | x | x |  |  |  | x | x |

**4. Sensitivity Analysis**

For convenience the main results of the compositional linear regression models are shown here:

| Cardio-metabolic Risk Marker | Retained ilr-coordinate | Regression Coefficient | Coefficient of Determination (R^2^) | LRT p-value for overall significance of waking day composition |
| --- | --- | --- | --- | --- |
| BMI | $\frac{1}{\sqrt{2}}\ln\frac{SB}{MVPA}$ | 1.374 | 0.1817 | <0.001 |
| Waist circumference | $\frac{1}{\sqrt{2}}\ln\frac{SB}{MVPA}$ | 3.601 | 0.3152 | <0.001 |
| Waist-to-hips ratio | $\frac{1}{\sqrt{2}}\ln\frac{SB}{MVPA}$ | 0.011 | 0.5034 | <0.001 |
| HDL | $\frac{1}{\sqrt{2}}\ln\frac{SB}{MVPA}$ | -0.050 | 0.2492 | <0.001 |
| Total cholesterol | None | n/a | n/a | 0.090 |
| Systolic blood pressure | None | n/a | n/a | 0.467 |
| Diastolic blood pressure | None | n/a | n/a | 0.199 |
| VO_2_ maximum | $\sqrt{\frac{2}{3}}\ln\frac{SB}{\sqrt{MVPA LIPA}}$ | -0.075 | 0.3708 | <0.001 |

The comparable results after eliminating a random 10% of the population are shown here:

| Cardio-metabolic Risk Marker | Retained ilr-coordinate | Regression Coefficient | | Coefficient of Determination (R^2^) | LRT p-value for overall significance of waking day composition |
| --- | --- | --- | --- | --- | --- |
| BMI | $\frac{1}{\sqrt{2}}\ln\frac{SB}{MVPA}$ | | 1.412 | 0.1873 | <0.001 |
| Waist circumference | $\frac{1}{\sqrt{2}}\ln\frac{SB}{MVPA}$ | | 3.745 | 0.3218 | <0.001 |
| Waist-to-hips ratio | $\frac{1}{\sqrt{2}}\ln\frac{SB}{MVPA}$ | | 0.012 | 0.5041 | <0.001 |
| HDL | $\frac{1}{\sqrt{2}}\ln\frac{SB}{MVPA}$ | | -0.054 | 0.2563 | <0.001 |
| Total cholesterol | None | | n/a | n/a | 0.079 |
| Systolic blood pressure | None | | n/a | n/a | 0.561 |
| Diastolic blood pressure | None | | n/a | n/a | 0.251 |
| VO_2_ maximum | $\sqrt{\frac{2}{3}}\ln\frac{SB}{\sqrt{MVPA LIPA}}$ | | -0.074 | 0.3803 | <0.001 |

**5. Codes**

R Codes used in the production of these results will be made available on http://www.opencoda.net.
